# Delayed surgery is associated with adverse outcomes in patients with hip fracture undergoing hip arthroplasty

**DOI:** 10.1186/s12891-023-06396-9

**Published:** 2023-04-13

**Authors:** Shencai Liu, Li Qiang, Qinfeng Yang, Lei Fan, Jian Wang, Yusheng Yang, Zhanjun Shi, Tao Li

**Affiliations:** 1 Department of Orthopedics, Nanfang Hospital, Southern Medical University, Guangzhou, 510515 Guangdong Province China; 2 Department of Joint Surgery, The Second Affiliated Hospital of Hainan Medical College, Hainan, 570000 China; 3 Department of Orthopaedics, Division of Orthopaedics and Traumatology, Nanfang Hospital, Southern Medical University, Guangzhou, 510515 Guangdong Province China

**Keywords:** Hip fracture, Arthroplasty, Surgery time, Complications

## Abstract

**Background:**

Hip arthroplasty (HA) is one of the most effective procedures for patients with hip fractures. The timing of surgery played a significant role in the short-term outcome for these patients, but conflicting evidence has been found.

**Methods:**

The Nationwide Inpatient Sample database was investigated from 2002 to 2014 and identified 247,377 patients with hip fractures undergoing HA. The sample was stratified into ultra-early (0 day), early (1–2 days) and delayed (3–14 days) groups based on time to surgery. Yearly trends, postoperative surgical and medical complications, postoperative length of hospital stay (POS) and total costs were compared after propensity scores were matched between groups by demographics and comorbidity.

**Results:**

From 2002 to 2014, the percentage of hip fracture patients who underwent HA increased from 30.61 to 31.98%. Early surgery groups showed fewer medical complications but higher surgical complications. However, specific complication evaluation showed both ultra-early and early groups decreased most of the surgery and medical complications with increasing post hemorrhagic anemia and fever. Medical complications were also reduced in the ultra-early group, but surgical complications increased. Early surgery groups reduced the POS by 0.90 to 1.05 days and total hospital charges by 32.6 to 44.9 percent than delayed surgery groups. Ultra-early surgery showed no benefit from POS than early group, but reduced total hospital charges by 12.2 percent.

**Conclusion:**

HA surgery performed within 2 days showed more beneficial effects on adverse events than delayed surgery. But surgeons should be cognizant of the potential increased risks of mechanical complications and post-hemorrhagic anemia.

**Supplementary Information:**

The online version contains supplementary material available at 10.1186/s12891-023-06396-9.

## Introduction

Hip fracture is one of the most serious health problems encountered by health care providers and patients. It was reported associated with a high mortality rate of 7–10% and was among the top 10 causes of disability globally [1–4]. Worldwide, 4.5 million people are disabled by a hip fracture each year. The estimated annual health care costs could reach $9.8 billion in the United States by the year 2040 [4, 5]. Fortunately, both internal fixation and arthroplasty (total hip arthroplasty or hemiarthroplasty) have demonstrated to be effective for the treatment of this condition. The outcome of a femoral fracture is influenced by a number of factors, including the age of the patient, gender, comorbidities, and status on anticoagulation therapy. This is in addition to the general health of the patient [6]. Prognosis may be influenced by the timing of surgery. In many studies, early surgery has been shown to increase the risk of perioperative complications. These complications include pneumonia, deep vein thrombosis, bleeding, pulmonary embolism, urinary tract infection, and decubital ulcers.

However, the short- and long-term outcomes, and the perioperative complications, of early surgery remain controversial. In addition, the optimal cut-off time for early surgery remains unclear. Recently, a large database study showed that hip arthroplasty for hip fracture was associated with higher risk of postoperative complications and decreased likelihood of discharge home, compared with osteoarthritis [7]. However, one of the main reasons might be the prolonged time from hospital admission to surgery in hip fracture patients.

Actually, guidelines recommend that hip fracture surgery be performed within 48 h or even as little as 6 h after the event [8, 9]. However, surgery was more likely to be delayed in patients who were sick on admission. Currently, the appropriate time for surgery is still unclear and controversial evidence was presented. A systematic review of 52 published studies involving 291,413 patients. The results showed a less likely beneficial effect of early surgery, especially in relation to mortality [10]. But a recent study that included 42,230 hip fracture patients from 72 hospitals over 5 years showed differently. The results showed patients who received surgery after 24 h had a significantly higher risk of 30-day mortality (6.5% vs 5.8%; 95%CI, 0.23–1.35) and the composite outcome (12.2% vs 10.1%; 95%CI, 1.43–2.89) [11].

Since some of the previous studies used small sample cohorts, few investigated complications, and were not specific to arthroplasty procedures, it is still unclear how surgical delay affects the outcome of arthroplasty. To the extent of our knowledge, there was no large-scale database investigation about this topic reported. We investigated the Nationwide Inpatient Sample (NIS) database to explore the influence of time to surgery on arthroplasty postoperative complications.

## Materials and methods

### Data source

The data source for this study was the NIS database, which is part of the Healthcare Cost and Utilization Project, Agency for Healthcare Research and Quality and is the largest all-payer database of inpatient admissions in the United States. Each year, the NIS collects a stratified sample of 20% of hospitalizations from more than 1000 hospitals in the United States. The available data include demographics, diagnosis and procedure codes (defined by the International Classification of Diseases, 9^th^ edition (ICD-9)), insurance information, hospital information, length of stay (LOS), total charges and discharge position. Data is publicly available and de-identified. Therefore, this study was deemed exempt by the institutional review board.

### Study population

Patients who had a diagnosis of closed hip fracture defined by ICD-9 codes 820.0, 820.00, 820.01, 820.02, 820.03, 820.09, 820.2, 820.20, 820.21, 820.22, and 820.8 were identified between January 1, 2002 and December 31, 2014 (*n* = 887755). And then patients who had a main procedure for hip arthroplasty (both hemi- and total hip arthroplasty) were identified using the ICD-9 procedure codes 81.51 and 81.52 (*n* = 273963). Patients were excluded from this study if they were less than 18 years old, or transferred admission from another hospital, or had bilateral or revision hip arthroplasty, or pathologic fracture, or days from admission to procedure were less than 0 days and longer than 14 days (*n* = 26586). Based on the day after admission to the procedure, the samples were stratified into three groups: ultra-early group (less than 24 h, *n* = 56403), early group (1–2 days, *n* = 158254), and delayed group (3–14 days, *n* = 32715).

### Outcomes

The postoperative complications were identified by the ICD-9 diagnosis codes as described in previous studies [12–15]. 31 different kinds of postoperative complications were analyzed as shown in Table 1. When one or more surgical or medical complications occur, the term “any complication” is used.

**Table 1:** Postoperative complications and the ICD-9 diagnosis codes

Complications	ICD-9 diagnosis codes
Surgical complications	
Postoperative hemorrhagic anemia	285.1
Hematoma/seroma	719.15, 719.16, 729.92, 998.11–998.13
Wound infection	682.6, 682.9, 890.0, 890.1, 890.2, 894.0, 894.1, 894.2, 998.5, 998.51, 998.59, 998.83
Wound dehiscence	998.3, 998.31, 998.32, 998.33
Irrigation and debridement	86.04, 86.09, 86.22, 86.28, 86.3
Mechanical complication of implant	996.40, 996.41, 996.43–996.49, 996.76–996.79
Periprosthetic infection	996.66, 996.67, 996.69
Dislocation of prosthetic joint	835.00–835.03, 835.10–835.13, 996.42
Peripheral nerve injury	956.0–956.9
Medical complications	
Fever	780.60, 780.62
Sepsis	995.91, 995.92
Thrombocytopenia	287.40, 287.5
Postoperative shock	998.0
Altered mental status	780.97
Cognitive symptoms	799.5x
Postoperative delirium	293.0
Central nervous system	997.0x
Stroke	97.02
Acute myocardial infarction	410, 410.x, 997.1
Peripheral vascular	997.2
Pulmonary	997.3, 997.31, 997.32
Pulmonary insufficiency following surgery	518.51, 518.52, 518.53
Pneumonia	480–480.9, 481, 482–482.9, 483, 483.1, 483.8, 484, 484.1, 484.3, 484.5–484.8, 485, 486, 487, 507
Gastrointestinal	997.4
Genitourinary	584.1–584.4, 599.0
Urinary tract infection	599, 997.5
Acute renal failure	584.5–584.9
Pulmonary embolism	415.11, 415.13, 415.19
Deep venous thrombosis	451.11, 451.19, 451.2, 451.81, 453.40–453.42
Transfusion	99.03–99.05, 99.07

Total charges and the length of the postoperative stay were also analyzed. The total charges were collected from the database. The length of postoperative stay (POS) was the difference between LOS and the days from admission to procedure.

### Covariates

The covariates included patient comorbidities that were identified using the Charlson Comorbidity Score, which includes 16 comorbidities. The strategy described in previous studies was used in this study to assess the severity of these comorbidities [16, 17]. A specific point value was assigned to a comorbidity and patients with higher scores were considered to have more serious comorbidities. The comorbid conditions and their point values were as follows: myocardial infarction (1), congestive heart disease (1), peripheral vascular disease (1), cerebrovascular disease (1), dementia (1), chronic pulmonary disease (1), connective tissue disease (1), peptic ulcer disease (1), liver disease (1), diabetes without complications (1), diabetes with complications (2), hemiplegia (2), any malignancy without metastasis including leukemia and lymphoma (2), renal failure (3), metastatic solid tumor (6), and AIDS/HIV (6).

### Data analysis

All the data analysis was performed with IBM SPSS statistics 22.0. The *P* values were 2 tailed, and a value of less than 0.05 was used for statistical significance. Propensity score matching was performed to mitigate potential confounding variables such as age, gender, race, insurance, Charlson Comorbidity Score, admission day was weekend, and elective admission. As the strength of the association between exposure and outcome increased in the propensity score, the bias decreased. It is worth noting that empirical power increased with an increase in events per confounder in both techniques, however, the increase in propensity scores was more significant [18]. The matching was performed with a 1:2 ultra-early to early group ratio, a 1:1 ultra-early to delayed group ratio, and a 3:1 early to delayed group ratio. Student-t test was applied to compare continuous variables, and the Chi-square test for nominal and ordinal variables. Logistic regression was used to calculate the odds ratios (ORs) of any surgical and medical complications compared between two groups. Linear regression was used to calculate parameter estimates for mean POS and total charges, and percentage differences were calculated to describe the results of linear regression with the formula (e^b^-1) × 100, where b is the parameter estimate of a log-transformed dependent variable.

## Results

### Patient characteristics

**Fig. 1:**
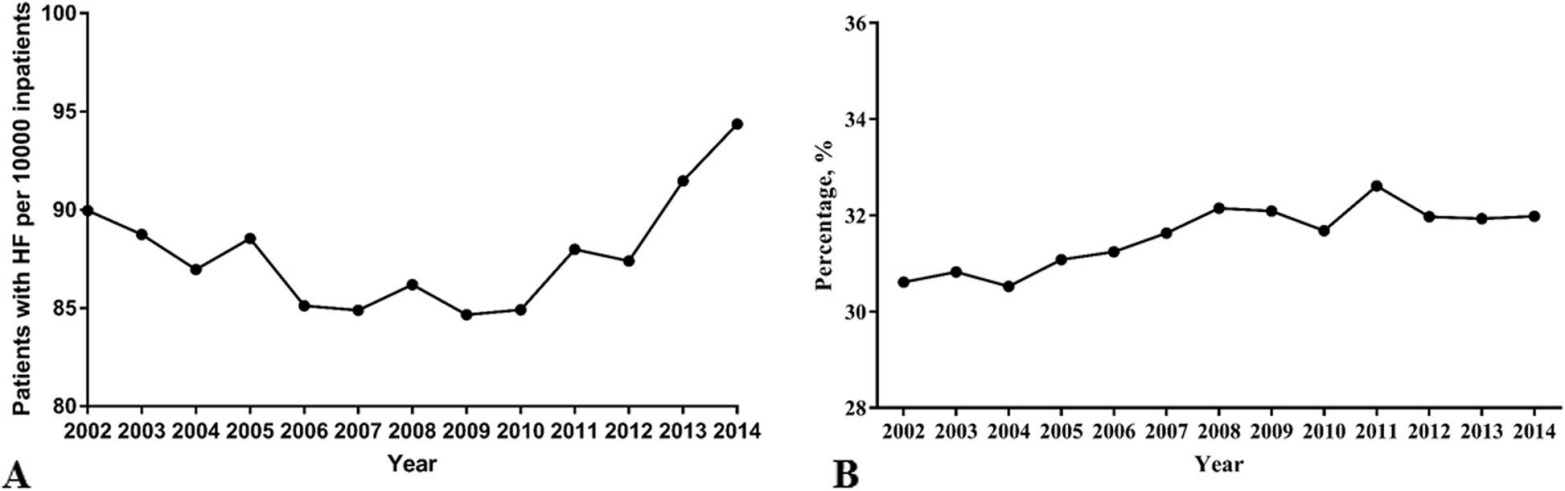
The annual number of patients with hip fracture (**A**) and the annual percentage of hip arthroplasty performed in hip fracture patients (**B**). HF: hip fracture

The annual occurrence of hip fracture was 0.90% in 2002 and 0.94% in 2014 (*P* < 0.001). The other annual occurrences were shown in Fig. 1, indicating a U-type tendency. The percentage of patients undergoing hip arthroplasty was 30.61% in 2002 and 31.98% in 2014 (*P* < 0.001), which indicated a slight increase (Fig. 1). The patient characteristics showed significant heterogeneity (Table 2). After propensity score matching, the demographics were presented in Tables 3, 4, 5.

**Table 2:** Demographics of study population

	Ultra-early	Early	Delayed	*P* value
N	56,408	158,254	32,715	
Age, mean ± SD	79.82 ± 10.32	80.63 ± 9.59	79.72 ± 10.26	< 0.001
Age stratification, in years old, n (%)				
18–39	150 (0.3)	190 (0.1)	102 (0.3)	< 0.001
40–49	548 (1.0)	947 (0.6)	314 (1.0)	
50–59	2514 (4.5)	5510 (3.5)	1454 (4.4)	
60–69	6352 (11.3)	15,684 (9.9)	3500 (10.7)	
70–79	15,126 (26.8)	42,440 (26.8)	9019 (27.6)	
80–89	26,712 (47.4)	79,358 (50.1)	15,603 (47.7)	
> = 90	5006 (8.9)	14,125 (8.9)	2723 (8.3)	
Gender, n (%)				
Female	41,817 (74.1)	114,900 (72.6)	21,410 (65.4)	< 0.001
Male	14,577 (25.8)	43,318 (27.4)	11,303 (34.5)	
Missing	14 (0.0)	36 (0.0)	2 (0.0)	
Race, n (%)				
White	44,039 (78.1)	124,825 (78.9)	24,694 (75.5)	< 0.001
Non-white	4843 (8.6)	16,248 (10.3)	4929 (15.1)	
Missing	7526 (13.3)	17,181 (10.9)	3092 (9.5)	
Insurance, n (%)				
Medicare	48,223 (85.5)	138,236 (87.4)	28,463 (87.0)	< 0.001
Medicaid	889 (1.6)	2739 (1.7)	984 (3.0)	
Private	5727 (10.2)	13,287 (8.4)	2429 (7.4)	
Self-pay	521 (0.9)	1361 (0.9)	330 (1.0)	
No charge	42 (0.1)	154 (0.1)	41 (0.1)	
Other	910 (1.6)	2262 (1.4)	423 (1.3)	
Missing	96 (0.2)	215 (0.1)	45 (0.1)	
Charlson Comorbidity Score, n (%)				
0	21,955 (38.9)	54,812 (34.6)	7933 (24.2)	< 0.001
1	17,997 (31.9)	50,084 (31.6)	9901 (30.3)	
2	7139 (12.7)	21,939 (13.9)	5649 (17.3)	
> = 3	8905 (15.8)	29,889 (18.9)	8922 (27.3)	
Missing	412 (0.7)	1530 (1.0)	310 (0.9)	
Mean ± SD	1.24 ± 1.55	1.41 ± 1.64	1.87 ± 1.86	< 0.001
Weekend admission, n (%)				
No	42,942 (76.1)	115,921 (73.2)	23,551 (72.0)	< 0.001
Yes	13,466 (23.9)	42,333 (26.8)	9164 (28.0)	
Elective admission, n (%)				
No	50,082 (88.8)	150,211 (94.9)	31,015 (94.8)	< 0.001
Yes	6209 (11.0)	7774 (4.9)	1639 (5.0)	
Missing	117 (0.2)	269 (0.2)	61 (0.2)

**Table 3:** Demographics of matched pairs (1:2) in the study population

	Matched pairs (1:2)		
	Ultra-Early	Matched Early	*P* value
N	52,374	104,748	
Age stratification, in years old, n (%)			
18–39	63 (0.1)	153 (0.1)	0.057
40–49	341 (0.7)	790 (0.8)	
50–59	1866 (3.6)	4189 (4.0)	
60–69	5413 (10.3)	11,715 (11.2)	
70–79	14,109 (26.9)	27,602 (26.4)	
80–89	25,740 (49.1)	50,088 (47.8)	
> = 90	4842 (9.2)	10,211 (9.7)	
Gender, n (%)			
Male	13,474 (25.7)	27,378 (26.1)	0.185
Female	38,886 (74.2)	77,347 (73.8)	
Missing	14 (0.0)	23 (0.0)	
Race, n (%)			
White	41,289 (78.8)	82,198 (78.5)	0.160
Non-white	4535 (8.7)	10,288 (9.8)	
Missing	6550 (12.5)	12,262 (11.7)	
Insurance, n (%)			
Medicare	45,586 (87.0)	90,540 (86.4)	0.085
Medicaid	793 (1.5)	1783 (1.7)	
Private	4625 (8.8)	9614 (9.2)	
Self-pay	469 (0.9)	942 (0.9)	
No charge	41 (0.1)	86 (0.1)	
Other	783 (1.5)	1620 (1.5)	
Missing	77 (0.1)	163 (0.2)	
Charlson Comorbidity Score, n (%)			
0	19,491 (37.2)	38,975 (37.2)	0.085
1	16,874 (32.2)	33,203 (31.7)	
2	6889 (13.2)	13,917 (13.3)	
> = 3	8713 (16.6)	17,755 (17.0)	
Missing	407 (0.8)	898 (0.9)	
Weekend admission, n (%)			
No	39,255 (75.0)	76,686 (73.2)	0.074
Yes	13,119 25.0)	28,062 (26.8)	
Elective admission, n (%)			
No	50,068 (95.6)	100,136 (95.6)	1
Yes	2204 (4.2)	4408 (4.2)	
Missing	102 (0.2)	204 (0.2)

**Table 4:** Demographics of matched pairs (1:1) in the study population

	Matched pairs (1:1)		
	Ultra-Early	Matched Delayed	*P* value
N	31,418	31,418	
Age stratification, in years old, n (%)			
18–39	79 (0.3)	88 (0.3)	0.082
40–49	249 (0.8)	295 (0.9)	
50–59	1211 (3.9)	1322 (4.2)	
60–69	3205 (10.2)	3339 (10.6)	
70–79	8768 (27.9)	8628 (27.5)	
80–89	15,214 (48.4)	15,081 (48.0)	
> = 90	2692 (8.6)	2665 (8.5)	
Gender, n (%)			
Male	10,257 (32.6)	10,384 (33.1)	0.559
Female	21,159 (67.3)	21,032 (66.9)	
Missing	2 (0.0)	2 (0.0)	
Race, n (%)			
White	24,602 (78.3)	24,343 (77.5)	0.214
Non-white	3750 (11.9)	3985 (12.7)	
Missing	3066 (9.8)	3090 (9.8)	
Insurance, n (%)			
Medicare	27,640 (88.0)	27,458 (87.4)	0.102
Medicaid	685 (2.2)	760 (2.4)	
Private	2313 (7.4)	2407 (7.7)	
Self-pay	331 (1.1)	302 (1.0)	
No charge	34 (0.1)	34 (0.1)	
Other	369 (1.2)	415 (1.3)	
Missing	46 (0.1)	42 (0.1)	
Charlson Comorbidity Score, n (%)			
0	7731 (24.6)	7928 (25.2)	0.222
1	10,057 (32.0)	9813 (31.2)	
2	5483 (17.5)	5472 (17.4)	
> = 3	7843 (25.0)	7896 (25.1)	
Missing	304 (1.0)	309 (1.0)	
Weekend admission, n (%)			
No	23,695 (75.4)	22,643 (72.1)	0.063
Yes	7723 (24.6)	8775 (27.9)	
Elective admission, n (%)			
No	29,722 (94.6)	29,722 (94.6)	1
Yes	1638 (5.2)	1638 (5.2)	
Missing	58 (0.2)	58 (0.2)

**Table 5:** Demographics of matched pairs (3:1) in the study population

	Matched pairs (3:1)		
	Early	Matched Delayed	*P* value
N	94,356	31,452	
Age stratification, in years old, n (%)			
18–39	73 (0.1)	36 (0.1)	0.085
40–49	599 (0.6)	225 (0.7)	
50–59	3507 (3.7)	1179 (3.7)	
60–69	9413 (10.0)	3257 (10.4)	
70–79	25,921 (27.5)	8714 (27.7)	
80–89	46,854 (49.7)	15,353 (48.8)	
> = 90	7989 (8.5)	2688 (8.5)	
Gender, n (%)			
Male	30,573 (32.4)	10,326 (32.8)	0.360
Female	63,779 (67.6)	21,125 (67.2)	
Missing	4 (0.0)	1 (0.0)	
Race, n (%)			
White	74,439 (78.9)	24,492 (77.9)	0.189
Non-white	11,268 (11.9)	3878 (12.3)	
Missing	8649 (9.2)	3082 (9.8)	
Insurance, n (%)			
Medicare	83,845 (88.9)	27,671 (88.0)	0.132
Medicaid	1753 (1.9)	605 (1.9)	
Private	6496 (6.9)	2387 (7.6)	
Self-pay	849 (0.9)	296 (0.9)	
No charge	103 (0.1)	35 (0.1)	
Other	1179 (1.2)	416 (1.3)	
Missing	131 (0.1)	42 (0.1)	
Charlson Comorbidity Score, n (%)			
0	23,555 (25.0)	7916 (25.2)	0.351
1	29,798 (31.6)	9805 (31.2)	
2	16,244 (17.2)	5469 (17.4)	
> = 3	23,919 (25.3)	7952 (25.3)	
Missing	840 (0.9)	310 (1.0)	
Weekend admission, n (%)			
No	69,101 (73.2)	22,634 (72.0)	0.087
Yes	25,255 (26.8)	8818 (28.0)	
Elective admission, n (%)			
No	89,658 (95.0)	29,886 (95.0)	1
Yes	4557 (4.8)	1519 (4.8)	
Missing	141 (0.1)	47 (0.1)	

### Postoperative surgical and medical complications

The risk of any postoperative complications was lower in the ultra-early group than in the early group (48.1% vs. 49.9%), with an OR of 0.961 (95% CI 0.946–0.975, *P* < 0.001) (Additional file 1: Table S1). Similarly, the risk of any postoperative complications was decreased in the ultra-early group than in the delayed group (49.5% vs. 60.0%), with an OR of 0.821 (95% CI 0.804–0.839, *P* < 0.001) (Additional file 1: Table 1). The above data indicate that the ultra-early group had the lowest risk of any postoperative complications (Additional file 1: Table 1). Any medical complications showed a similar tendency. However, the risk of any surgical complications was higher in the ultra-early group than in the early group (29.4% vs. 27.1%), with an OR of 1.086 (95% CI 1.065–1.107, *P* < 0.001) (Additional file 1: Table 1). Similarly, the risk of any surgical complications was increased in the ultra-early group than in the early group (30.0% vs. 23.5%), with an OR of 1.227 (95% CI 1.234–1.312, *P* < 0.001) (Additional file 1: Table S1). The above data indicate that the ultra-early group had the highest risk of any surgical complications. The specific surgical complications were shown in Additional file 2: Table S2, Additional file 3: Table S3 and Additional file 4: Table S4, and the specific medical complications were shown in Additional file 5: Table S5,  Additional file 6: Table S6 and Additional file 7: Table S7.

After multivariate logistic regression, delayed surgery was found as an independent risk factor for wound infection and pulmonary embolism (Additional file 2: Table S2, Additional file 3: Table S3 and Additional file 4: Table S4). In addition, early surgery and delayed surgery were found as independent risk factors for pulmonary embolism (Additional file 5: Table S5,  Additional file 6: Table S6 and Additional file 7: Table S7).

### Lengths of postoperative stay

The mean POS in the delayed group was the longest among the three groups, which was longer than both the ultra-early group (5.99 ± 5.79 vs. 5.09 ± 3.50, *P* < 0.001) and the early group (5.97 ± 5.75 vs. 4.92 ± 3.90, *P* < 0.001). The early group showed a slightly shorter mean POS than the ultra-early group (4.77 ± 3.59 vs. 4.92 ± 3.21, *P* < 0.001). After linear regression analysis, the largest difference was shown between the early group and the matched delayed group. The early group showed an average difference of 1.05 days (95% CI 0.99–1.11, *P* < 0.001) shorter and a percent difference of 15.0% (95% CI 11.6%-15.7%, *P* < 0.001) less than the postponement group (Additional file 8: Table S8 and Additional file 9: Table S9).

### Total hospital charges

The mean total charges were less in the ultra-early group than in the early group (44.75 ± 34.00 vs. 50.88 ± 38.59, *P* < 0.001). Additionally, the mean total charges were less in the ultra-early group than in the delayed group (46.65 ± 35.24 vs. 76.42 ± 68.52, *P* < 0.001). Based on the above results, the ultra-early group had the lowest average total charges. Furthermore, the early group showed a lower average total charge than the delayed group (52.86 ± 40.67 vs. 76.30 ± 68.58, *P* < 0.001). After linear regression analysis, the maximum difference was found between the ultra-early group and the matched delayed group. Meanwhile, the ultra-early group showed a mean difference of 29.67 × 103 dollars (95% CI 28.81–30.53, *P* < 0.001) less and 44.9% less than the delayed group (95% CI 43.9%-45.9%, *P* < 0.001) (Additional file 8: Table S8 and Additional file 9: Table S9).

## Discussion

Hip fracture is a common event in the elderly, leading to substantial morbidity and mortality. Surgical timing is a critical issue for these patients. While early surgery might be associated with less suffering, delayed surgery allows sufficient time for physiological stabilization. The details of surgical timing were still unclear, despite guidelines suggesting surgery within 48 h. Herein, this study aimed to explore the influence of time to surgery on postoperative complications following arthroplasty based on the NIS database.

Many studies investigated the relationship between surgical timing and mortality. In addition to being important for assessing injury and surgery, mortality may not be an ideal indicator of surgical timing. Theoretically, mortality was more likely to be associated with health status before injury, the specific type of fracture and the specific type of procedure, other than surgical timing. Thus, conflicting evidence might be yielded. In the systemic analysis of 52 related studies, 22 studies reported reduced mortality for early surgery, 2 studies reported increased mortality, and 25 studies reported no difference [10].

We hypothesized that surgical timing was more likely to be related to postoperative complications, postoperative hospital stay and total hospital charges. In concern of surgical complications, the results indicated that early surgery (both ultra-early group and early group) reduced the risk of wound infection, mechanical complications, wound dehiscence, periprosthetic infection and dislocation. However, an increased risk of post hemorrhagic anemia was shown in early surgery. No difference was observed in terms of irrigation and debridement, and nerve injury. In concern of medical complications, the results showed that early surgery (both ultra-early group and early group) reduced the risk of sepsis, pneumonia, genitourinary, acute renal failure, pulmonary embolism, deep venous thrombosis, altered mental status, postoperative delirium, and gastrointestinal. However, an increased risk of fever was shown in early surgery. No difference was observed in others. Based on the above data, hip arthroplasty surgery within 48 h for hip fracture patients can reduce surgical and medical complications. However, attention should be paid to the increase in postoperative hemorrhagic anemia and fever. The results supported the former studies of Leer-Salvesen et al., which was also a database investigation of the Norwegian Patient Registry and included 83,727 patients over 10 years [19]. However, conflicting evidence yielded from the studies of Craik et al. [20] and Lim et al. [21], both of which investigated also an over ten-year period data resulting in no significant differences in regard of complications. The reasons of controversial evidences might be multiple aspects. Firstly, most of the former studies were cohorts from small samples. The largest cohort in the study of Bottle A et al. included 129,522 patients [22]. In our study, we used national data over a period of 13 years, which resulted in 247,377 patients, which yielded stronger results. Secondly, most of the previous studies ignored the influence of different surgical procedures on complications. In these studies, both osteosynthesis and replacement surgeries were analyzed for complications. Our study adopted only hip arthroplasty as a single type of surgical procedure for the management of hip fracture, which minimized the confounding factors. Furthermore, this study performed propensity score matching to mitigate the potential confounding variables (age, gender, race, insurance, Charlson Comorbidity Score, admission day was weekend, and elective admission), making the results scientific. Thirdly, most of the earlier studies had inadequate data covering parts of the possible complications. Our study covered 31 of the most common postoperative complications.

Another critical issue was the cutoff time for “early surgery”. Different published studies defined different cutoffs. In the studies by Leer-Salvesen et al. [19] and Shiga et al. [23], 48 h was thought to be an appropriate cutoff. But the studies by Zajonz et al. [24] and Majumdar et al. [25] recommended surgery within 24 h. And the study by Uzoigwe et al. even suggested surgery within 12 h [26]. To the extent of our knowledge, most studies have tended to accept surgery within 48 h as early surgery, which was also recommended by the guidelines [3, 8]. Our study adopted this cutoff as well. The results of our study showed that ultra-early surgery (within 24 h) increased the risk of post hemorrhagic anemia and mechanical complications, indicating no beneficial effect on surgical complications. Nevertheless, ultra-early surgery reduced the risk of medical complications, including fever, sepsis, thrombocytopenia, myocardial infarction, pneumonia, genitourinary, acute renal failure, pulmonary embolism and deep venous thrombosis.

The results of multivariate logistic regression showed that time of surgery was an independent risk factor for wound infection and pulmonary embolism. To the extent of our knowledge, this was the first time this was reported. A strictly designed study was needed to confirm this finding.

POS and total hospital charges were incorporated into our study to assess resource utilization. Former studies showed longer LOS in delayed surgery from 1.9 days to 6 days [27, 28]. Conflicting results were also reported in the study by Sellan et al. [29], which showed no effect. However, these results might be misleading, because the difference in LOS might be generated from preoperative stay. Different from previous studies, we calculated the indicator POS instead of LOS to generate an accurate relationship between surgical time and hospital stay. The results indicated that early surgery reduced POS by 0.90 to 1.05 days than delayed surgery, about 9.3 to 15.0 percent less. Based on the POS calculated differences between LOS and preoperative hospital stay, the LOS would be more significant than revealed by this study. Thus, the results of our study supported former studies and confirmed that the LOS difference was also generated from POS.

Different results appeared in comparison of ultra-early and early surgery. The results indicated ultra-early surgery increased POS by 0.15 days than early surgery, about 5.7 percent longer. In a previous study by Majumdar et al., LOS was found to work in favour of ultra-early surgery. In considering the distinction between POS and LOS, we suggested our study reveal a more accurate relationship of ultra-early and early surgery [25].

The total hospital charges in our study showed a tendency for surgery to be performed earlier and cost less. Compared to delayed surgery, early surgery reduced total hospital charges by 32.6 to 44.9%, and ultra-early surgery reduced 12.2%. The results were consistent with the former study [30].

Several limitations of this study should be noted. First, the administrative information was based on ICD-9 coding, which was prone to errors. Second, given the limitations of ICD-9 coding, we cannot distinguish hemi-hip arthroplasty from total hip arthroplasty. Although both procedures shared most of the characteristics, they were different in details. Further studies will have to account for the differences between hemi- and total hip arthroplasty as the latter is becoming more common. Third, the NIS database collected only the data of inpatients related to the hospital stay. Therefore, we were unable to investigate the mid-term and long-term outcomes that occurred after hospital discharge. Nevertheless, to the point of the authors’ knowledge, this is the only study reporting on the short-term outcomes of hip fracture patients who underwent hip arthroplasty at different surgical times. And these results are valuable as they demonstrate the associated adverse events that may occur.

## Conclusion

Based on the data of this study, we recommend hip arthroplasty surgery performed in hip fracture patients as soon as 48 h. This was to avoid the surgical and medical complications with an alert of increasing post hemorrhagic anemia and fever. Additionally, ultra-early surgery (within 24 h) reduced medical complications, but increased surgical complications, specifically hemorrhagic anemia and mechanical complications. Early surgery reduced the POS by 0.90 to 1.05 days and total hospital charges by 32.6 to 44.9 percent than delayed surgery. Ultra-early surgery showed no beneficial effect on POS than early, but decreased total hospital charges by 12.2 percent. Overall, early surgery showed more beneficial effects on adverse events than delayed surgery, and ultra-early surgery showed even slightly better outcomes.

## Supplementary Information

**Additional file 1:** **Table S1.** Logistic regression analysis ofperioperative complications.

**Additional file 2:** **Table S2.** Surgical complications of ultra-earlygroup and matched delayed group.

**Additional file 3:** **Table S3.** Surgical complications of early groupand matched delayed group.

**Additional file 4:** **Table S4.** Surgical complications of ultra-earlygroup and matched early group.

**Additional file 5: Table S5.** Medical complications of ultra-earlygroup and matched delayed group.

**Additional file 6:** **Table S6.** Medical complications of early groupand matched delayed group.

**Additional file 7:** **Table S7.** Medical complications of ultra-earlygroup and matched early group.

**Additional file 8:** **Table S8.** Length of postoperative stay and totalcharges.

**Additional file 9:** **Table S9.** Linear regression analysis of length ofpostoperative stay and total charges.

## Data Availability

This study is based on data provided by Nationwide Inpatient Sample (NIS) database, part of the Healthcare Cost and Utilization Project, Agency for Healthcare Research and Quality. The NIS database is a large publicly available full-payer inpatient care database in the United States and the direct web link to the database is https://www.ahrq.gov/data/hcup/index.html. Therefore, individual or grouped data cannot be shared by the authors.

## Abbreviation

HA: Hip arthroplasty
POS: Length of postoperative stay
HIS: Nationwide Inpatient Sample
LOS: Length of stay
ICD-9: International Classification of Disease, 9^th^ edition
OR: Odds ratios
