# Supplementary material for: Delayed surgery is associated with adverse outcomes in patients with hip fracture undergoing hip arthroplasty
Source: BMC Musculoskelet Disord. 2023 Apr 13;24:286. doi: 10.1186/s12891-023-06396-9 (PMC10100473; doi:10.1186/s12891-023-06396-9)
Supplement: Supplementary file 1 — Additional file 1: Table S1. Logistic regression analysis ofperioperative complications. [file 12891_2023_6396_MOESM1_ESM.docx]

Additional file 1: Table S1 Logistic regression analysis of perioperative complications

| Outcomes | Ultra-early | Early | Delayed | P value | Odds Ratio (95% CI) | P value |
| --- | --- | --- | --- | --- | --- | --- |
| Any Surgery Complications | 29.4(15416) | 27.1(28353) | - | <0.001 | 1.09(1.07,1.11) | <0.001 |
|  | 30.0(9411) | - | 23.5(7377) | <0.001 | 1.27(1.23,1.31) | <0.001 |
|  | - | 27.5(25919) | 23.5(7388) | <0.001 | 1.17(1.14,1.20) | <0.001 |
| Any Medical Complications | 29.6(15507) | 34.0(35609) |  | <0.001 | 0.87(0.85,0.88) | <0.001 |
|  | 31.4(9856) | - | 49.5(15563) | <0.001 | 0.63(0.61,0.64) | <0.001 |
|  | - | 36.3(34253) | 49.7(15623) | <0.001 | 0.71(0.70,0.72) | <0.001 |
| Any Complications | 48.1(25167) | 49.9(52311) | - | <0.001 | 0.96(0.95,0.98) | <0.001 |
|  | 49.5(15540) | - | 60.0(18840) | <0.001 | 0.82(0.80,0.84) | <0.001 |
|  | - | 51.6(48717) | 60.1(18897) | <0.001 | 0.85(0.84,0.87) | <0.001 |

Comparation was carried out between each group and the matched group, which was based on propensity score matching. That was a 1:2 ultra-early to early group ratio, a 1:1 ultra-early to delayed group ratio, and a 3:1 early to delayed group ratio.
