# Supplementary material for: Delayed surgery is associated with adverse outcomes in patients with hip fracture undergoing hip arthroplasty
Source: BMC Musculoskelet Disord. 2023 Apr 13;24:286. doi: 10.1186/s12891-023-06396-9 (PMC10100473; doi:10.1186/s12891-023-06396-9)
Supplement: Supplementary file 2 — Additional file 2: Table S2. Surgical complications of ultra-earlygroup and matched delayed group. [file 12891_2023_6396_MOESM2_ESM.docx]

Additional file 2: Table S2 Surgical complications of ultra-early group and matched delayed group

| Parameter | Univariate analysis, %(n) | | |  | Multivariate logistic regression | |
| --- | --- | --- | --- | --- | --- | --- |
|  | Ultra-early | Matched Delayed | P value |  | Odds Ratio (95% CI) | P value |
| Postoperative hemorrhagic anemia | 28.5(8966) | 20.7(6503) | <0.001 |  | 1.37(1.33,1.42) | <0.001 |
| Hematoma | 1.4(436) | 1.5(482) | 0.126 |  | - | - |
| Wound infection | 0.7(209) | 1.6(516) | <0.001 |  | 0.41(0.35,0.48)^a^ | <0.001 |
| Wound dehiscence | 0.0(12) | 0.1(25) | 0.033 |  | 0.48(0.24,0.96) | 0.037 |
| Irrigation and debridement | 0.0(0) | 0.0(0) | - |  | - | - |
| Mechanical complication | 0.7(220) | 1.1(344) | <0.001 |  | 0.61(0.51,0.73) | <0.001 |
| Periprosthetic infection | 0.1(18) | 0.2(49) | <0.001 |  | 0.37(0.21,0.63)^a^ | <0.001 |
| Dislocation | 0.2(64) | 0.3(94) | 0.017 |  | 0.68(0.50,0.94) | 0.018 |
| Nerve injury | 0.0(9) | 0.0(9) | 1 |  | - | - |

Comparation was carried out between ultra-early group and the matched delayed group, which was based on propensity score matching. That was a 1:1 ultra-early to delayed group ratio. a: independent risk factor.
