# Supplementary material for: Delayed surgery is associated with adverse outcomes in patients with hip fracture undergoing hip arthroplasty
Source: BMC Musculoskelet Disord. 2023 Apr 13;24:286. doi: 10.1186/s12891-023-06396-9 (PMC10100473; doi:10.1186/s12891-023-06396-9)
Supplement: Supplementary file 3 — Additional file 3: Table S3. Surgical complications of early groupand matched delayed group. [file 12891_2023_6396_MOESM3_ESM.docx]

Additional file 3: Table S3 Surgical complications of early group and matched delayed group

| Parameter | Univariate analysis, %(n) | | |  | Multivariate logistic regression | |
| --- | --- | --- | --- | --- | --- | --- |
|  | Early | Matched Delayed | P value |  | Odds Ratio (95% CI) | P value |
| Postoperative hemorrhagic anemia | 26.1(24660) | 20.8(6527) | <0.001 |  | 1.25(1.22,1.29) | <0.001 |
| Hematoma | 1.3(1185) | 1.5(473) | 0.001 |  | 0.84(0.75,0.93) | 0.001 |
| Wound infection | 0.7(657) | 1.6(516) | <0.001 |  | 0.43(0.38,0.48)^a^ | <0.001 |
| Wound dehiscence | 0.0(28) | 0.1(23) | 0.001 |  | 0.41(0.23,0.70)^a^ | 0.001 |
| Irrigation and debridement | 0.0(0) | 0.0(0) | - |  | - | - |
| Mechanical complication | 0.5(444) | 1.1(333) | <0.001 |  | 0.44(0.39,0.51) | <0.001 |
| Periprosthetic infection | 0.1(55) | 0.2(48) | <0.001 |  | 0.38(0.25,0.58) | <0.001 |
| Dislocation | 0.2(194) | 0.3(96) | 0.001 |  | 0.72(0.55,0.93) | 0.012 |
| Nerve injury | 0.0(26) | 0.0(8) | 0.843 |  | - | - |

Comparation was carried out between early group and the matched delayed group, which was based on propensity score matching. That was a 3:1 early to delayed group ratio. a: independent risk factor.
