# Supplementary material for: Delayed surgery is associated with adverse outcomes in patients with hip fracture undergoing hip arthroplasty
Source: BMC Musculoskelet Disord. 2023 Apr 13;24:286. doi: 10.1186/s12891-023-06396-9 (PMC10100473; doi:10.1186/s12891-023-06396-9)
Supplement: Supplementary file 4 — Additional file 4: Table S4. Surgical complications of ultra-earlygroup and matched early group. [file 12891_2023_6396_MOESM4_ESM.docx]

Additional file 4: Table S4 Surgical complications of ultra-early group and matched early group

| Parameter | Univariate analysis, % (n) | | |  | Multivariate logistic regression | |
| --- | --- | --- | --- | --- | --- | --- |
|  | Ultra-early | Matched Early | P value |  | Odds Ratio (95% CI) | P value |
| Postoperative Hemorrhagic Anemia | 28.2 (14760) | 25.8 (27060) | <0.001 |  | 1.09 (1.07,1.11) | <0.001 |
| Hematoma | 1.3 (659) | 1.2 (1267) | 0.409 |  | - | - |
| Wound infection | 0.6 (300) | 0.7 (698) | 0.029 |  | 0.87 (0.76,1.00)^a^ | 0.057 |
| Wound dehiscence | 0.0 (17) | 0.0 (32) | 0.875 |  | - | - |
| Irrigation and debridement | 0.0 (0) | 0.0 (0) |  |  | - | - |
| Mechanical complication | 0.6 (337) | 0.4 (461) | <0.001 |  | 1.47 (1.27,1.70) | <0.001 |
| Periprosthetic infection | 0.1 (33) | 0.1 (53) | 0.340 |  | - | - |
| Dislocation | 0.2 (96) | 0.2 (196) | 0.849 |  | - | - |
| Nerve injury | 0.0 (14) | 0.0 (23) | 0.565 |  | - | - |

Comparation was carried out between ultra-early group and the matched early group, which was based on propensity score matching. That was a 1:2 ultra-early to early group ratio. a: independent risk factor.
