# Supplementary material for: Delayed surgery is associated with adverse outcomes in patients with hip fracture undergoing hip arthroplasty
Source: BMC Musculoskelet Disord. 2023 Apr 13;24:286. doi: 10.1186/s12891-023-06396-9 (PMC10100473; doi:10.1186/s12891-023-06396-9)
Supplement: Supplementary file 5 — Additional file 5: Table S5. Medical complications of ultra-earlygroup and matched delayed group. [file 12891_2023_6396_MOESM5_ESM.docx]

Additional file 5: Table S5 Medical complications of ultra-early group and matched delayed group

| Parameter | Univariate analysis, % (n) | | |  | Multivariate logistic regression | |
| --- | --- | --- | --- | --- | --- | --- |
|  | Ultra-early | Matched Delayed | P value |  | Odds Ratio (95% CI) | P value |
| Fever | 1.7 (535) | 1.4 (440) | 0.002 |  | 1.21 (1.06,1.39) | 0.006 |
| Sepsis | 0.8 (261) | 2.8 (887) | <0.001 |  | 0.29 (0.26,0.34)^a^ | <0.001 |
| Thrombocytopenia | 4.5 (1399) | 4.7 (1491) | 0.080 |  | - | - |
| Postoperative shock | 0.1 (30) | 0.1 (42) | 0.157 |  | - | - |
| Altered mental status | 0.6 (200) | 0.8 (252) | 0.014 |  | 0.79 (0.66,0.96)^a^ | 0.015 |
| Cognitive symptoms | 0.0 (3) | 0.0 (0) | 0.253^b^ |  | - | - |
| Postoperative delirium | 1.6 (489) | 1.9 (583) | 0.004 |  | 0.83 (0.74,0.94) | 0.004 |
| Central nervous system | 0.3 (95) | 0.2 (78) | 0.196 |  | - | - |
| Stroke | 0.0 (0) | 0.0 (0) | - |  | - | - |
| Myocardial infarction | 1.6 (513) | 1.7 (541) | 0.384 |  | - | - |
| Peripheral vascular | 0.1 (25) | 0.1 (41) | 0.049 |  | 0.61 (0.37,1.00) | 0.051 |
| Pulmonary | 1.0 (324) | 1.0 (312) | 0.632 |  | - | - |
| Pulmonary insufficiency | 0.6 (181) | 0.7 (230) | 0.015 |  | 0.80 (0.65,0.99) | 0.041 |
| Pneumonia | 4.3 (1344) | 10.4 (3274) | <0.001 |  | 0.40 (0.38,0.43) | <0.001 |
| Gastrointestinal | 0.5 (159) | 0.7 (210) | 0.008 |  | 0.76 (0.62,0.93) | 0.008 |
| Genitourinary | 14.3 (4483) | 26.8 (8420) | <0.001 |  | 0.52 (0.50,0.54) | <0.001 |
| Urinary tract infection | 0.8 (265) | 0.8 (254) | 0.628 |  | - | - |
| Acute renal failure | 7.2 (2252) | 12.7 (3983) | <0.001 |  | 0.55 (0.52,0.58) | <0.001 |
| Pulmonary embolism | 0.5 (160) | 1.5 (473) | <0.001 |  | 0.34 (0.28,0.41)^a^ | <0.001 |
| Deep venous thrombosis | 0.4 (123) | 1.5 (478) | <0.001 |  | 0.26 (0.21,0.31) | <0.001 |
| Transfusion | 0.0 (0) | 0.0 (0) | - |  | - | - |

Comparation was carried out between ultra-early group and the matched delayed group, which was based on propensity score matching. That was a 1:1 ultra-early to delayed group ratio. a: independent risk factor; b: Fisher’s exact test.
