# Supplementary material for: Delayed surgery is associated with adverse outcomes in patients with hip fracture undergoing hip arthroplasty
Source: BMC Musculoskelet Disord. 2023 Apr 13;24:286. doi: 10.1186/s12891-023-06396-9 (PMC10100473; doi:10.1186/s12891-023-06396-9)
Supplement: Supplementary file 6 — Additional file 6: Table S6. Medical complications of early groupand matched delayed group. [file 12891_2023_6396_MOESM6_ESM.docx]

Additional file 6: Table S6 Medical complications of early group and matched delayed group

| Parameter | Univariate analysis, % (n) | | | Multivariate logistic regression | |
| --- | --- | --- | --- | --- | --- |
|  | Early | Matched Delayed | P value | Odds Ratio (95% CI) | P value |
| Fever | 2.0 (1898) | 1.4 (441) | <0.001 | 1.45 (1.31,1.61) | <0.001 |
| Sepsis | 1.0 (941) | 2.8 (894) | <0.001 | 0.35 (0.32,0.38) | <0.001 |
| Thrombocytopenia | 4.8 (4540) | 4.8 (1500) | 0.761 | - | - |
| Postoperative shock | 0.1 (106) | 0.1 (44) | 0.220 | - | - |
| Altered mental status | 0.6 (606) | 0.8 (256) | 0.001 | 0.80 (0.68,0.93) | 0.003 |
| Cognitive symptoms | 0.0 (13) | 0.0 (0) | 0.037^b^ | 31.53 (0.15,6589.40) | 0.206 |
| Postoperative delirium | 1.7 (1605) | 1.9 (592) | 0.034 | 0.90 (0.82,0.99) | 0.026 |
| Central nervous system | 0.3 (249) | 0.3 (81) | 0.849 | - | - |
| Stroke | 0.0 (0) | 0.0 (0) | - | - | - |
| Myocardial infarction | 1.7 (1625) | 1.7 (546) | 0.871 | - | - |
| Peripheral vascular | 0.1 (91) | 0.1 (40) | 0.143 | - | - |
| Pulmonary | 1.1 (1006) | 1.0 (306) | 0.159 | - | - |
| Pulmonary insufficiency | 0.7 (624) | 0.7 (225) | 0.311 | - | - |
| Pneumonia | 4.7 (4388) | 10.4 (3275) | <0.001 | 0.44 (0.42,0.46) | <0.001 |
| Gastrointestinal | 0.6 (531) | 0.7 (215) | 0.016 | 0.82 (0.70,0.97) | 0.018 |
| Genitourinary | 18.0 (16970) | 26.8 (8443) | <0.001 | 0.64 (0.63,0.66) | <0.001 |
| Urinary tract infection | 0.9 (815) | 0.8 (257) | 0.436 | - | - |
| Acute renal failure | 8.3 (7819) | 12.8 (4028) | <0.001 | 0.63 (0.60,0.65) | <0.001 |
| Pulmonary embolism | 0.7 (666) | 1.5 (469) | <0.001 | 0.47 (0.42,0.53)^a^ | <0.001 |
| Deep venous thrombosis | 0.5 (509) | 1.5 (474) | <0.001 | 0.36 (0.31,0.40) | <0.001 |
| Transfusion | 0.0 (0) | 0.0 (0) | - | - | - |

Comparation was carried out between early group and the matched delayed group, which was based on propensity score matching. That was a 3:1 early to delayed group ratio. a: independent risk factor; b: Fisher’s exact test.
