# Supplementary material for: Delayed surgery is associated with adverse outcomes in patients with hip fracture undergoing hip arthroplasty
Source: BMC Musculoskelet Disord. 2023 Apr 13;24:286. doi: 10.1186/s12891-023-06396-9 (PMC10100473; doi:10.1186/s12891-023-06396-9)
Supplement: Supplementary file 7 — Additional file 7: Table S7. Medical complications of ultra-earlygroup and matched early group. [file 12891_2023_6396_MOESM7_ESM.docx]

Additional file 7: Table S7 Medical complications of ultra-early group and matched early group

| Parameter | Univariate analysis, %(n) | | | Multivariate logistic regression | |
| --- | --- | --- | --- | --- | --- |
|  | Ultra-early | Matched Early | P value | Odds Ratio (95% CI) | P value |
| Fever | 1.7 (893) | 1.9 (2011) | 0.003 | 0.91 (0.84,0.98) | 0.017 |
| Sepsis | 0.6 (334) | 0.8 (870) | <0.001 | 0.76 (0.67,0.87) | <0.001 |
| Thrombocytopenia | 4.0 (2098) | 4.4 (4602) | <0.001 | 0.92 (0.87,0.97) | 0.001 |
| Postoperative shock | 0.1 (45) | 0.1 (107) | 0.341 | - | - |
| Altered mental status | 0.5 (265) | 0.5 (550) | 0.619 | - | - |
| Cognitive symptoms | 0.0 (4) | 0.0 (16) | 0.328 | - | - |
| Postoperative delirium | 1.5 (804) | 1.5 (1578) | 0.657 | - | - |
| Central nervous system | 0.3 (144) | 0.2 (258) | 0.282 | - | - |
| Stroke | 0.0 (0) | 0.0 (0) | - | - | - |
| Myocardial infarction | 1.5 (801) | 1.7 (1750) | 0.038 | 0.91 (0.83,0.99) | 0.025 |
| Peripheral vascular | 0.1 (41) | 0.1 (103) | 0.229 | - | - |
| Pulmonary | 1.0 (522) | 1.0 (1080) | 0.520 | - | - |
| Pulmonary insufficiency | 0.5 (251) | 0.5 (557) | 0.169 | - | - |
| Pneumonia | 3.8 (1987) | 4.2 (4395) | <0.001 | 0.89 (0.85,0.94) | <0.001 |
| Gastrointestinal | 0.5 (256) | 0.5 (536) | 0.556 | - | - |
| Genitourinary | 14.3 (7504) | 17.7 (18548) | <0.001 | 0.80 (0.78,0.82) | <0.001 |
| Urinary tract infection | 0.8 (404) | 0.8 (846) | 0.446 | - | - |
| Acute renal failure | 5.7 (2979) | 6.5 (6854) | <0.001 | 0.87 (0.83,0.90) | <0.001 |
| Pulmonary embolism | 0.5 (265) | 0.7 (756) | <0.001 | 0.69 (0.60,0.79)^a^ | <0.001 |
| Deep venous thrombosis | 0.4 (194) | 0.5 (543) | <0.001 | 0.73 (0.62,0.87) | <0.001 |
| Transfusion | 0.0 (0) | 0.0 (0) | - | - | - |

Comparation was carried out between ultra-early group and the matched early group, which was based on propensity score matching. That was a 1:2 ultra-early to early group ratio. a: independent risk factor.
