# Supplementary material for: Delayed surgery is associated with adverse outcomes in patients with hip fracture undergoing hip arthroplasty
Source: BMC Musculoskelet Disord. 2023 Apr 13;24:286. doi: 10.1186/s12891-023-06396-9 (PMC10100473; doi:10.1186/s12891-023-06396-9)
Supplement: Supplementary file 8 — Additional file 8: Table S8. Length of postoperative stay and totalcharges. [file 12891_2023_6396_MOESM8_ESM.docx]

Additional file 8: Table S8 Length of postoperative stay and total charges

| Outcomes | Ultra-early | Early | Delayed | P value |
| --- | --- | --- | --- | --- |
| POS (days), mean±SD | 4.92±3.21 | 4.77±3.59 | - | <0.001 |
|  | 5.09±3.50 | - | 5.99±5.79 | <0.001 |
|  | - | 4.92±3.90 | 5.97±5.75 | <0.001 |
| Total charges, ×10^3^ dollars, mean±SD | 44.75±34.00 | 50.88±38.59 | - | <0.001 |
|  | 46.65±35.24 | - | 76.42±68.52 | <0.001 |
|  | - | 52.86±40.67 | 76.30±68.58 | <0.001 |

Comparation was carried out between each group and the matched group, which was based on propensity score matching. That was a 1:2 ultra-early to early group ratio, a 1:1 ultra-early to delayed group ratio, and a 3:1 early to delayed group ratio.
