# Supplementary material for: Delayed surgery is associated with adverse outcomes in patients with hip fracture undergoing hip arthroplasty
Source: BMC Musculoskelet Disord. 2023 Apr 13;24:286. doi: 10.1186/s12891-023-06396-9 (PMC10100473; doi:10.1186/s12891-023-06396-9)
Supplement: Supplementary file 9 — Additional file 9: Table S9. Linear regression analysis of length ofpostoperative stay and total charges. [file 12891_2023_6396_MOESM9_ESM.docx]

Additional file 9: Table S9 Linear regression analysis of length of postoperative stay and total charges

|  | Mean difference (95%CI) | P value | Percent difference,  % (95%CI) | P value |
| --- | --- | --- | --- | --- |
| POS, days | | | | |
| Ultra-Early Vs. Early | 0.15 (0.12,0.19) | <0.001 | 5.7 (5.2,6.2) | <0.001 |
| Ultra-Early Vs. Delayed | -0.90 (-0.97,-0.82) | <0.001 | -9.3 (-10.2,-8.4) | <0.001 |
| Early Vs. Delayed | -1.05 (-1.11,-0.99) | <0.001 | -15.0 (-15.7,-11.6) | <0.001 |
| Total charges,×10^3^ dollars | | | | |
| Ultra-Early Vs. Early | -5.97 (-6.37,-5.58) | <0.001 | -12.2 (-12.9,-11.6) | <0.001 |
| Ultra-Early Vs. Delayed | -29.67 (-30.53,-28.81) | <0.001 | -44.9 (-45.9,-43.9) | <0.001 |
| Early Vs. Delayed | -23.40 (-24.03,-22.78) | <0.001 | -32.6 (-33.4,-31.8) | <0.001 |

Comparation was carried out between each group and the matched group, which was based on propensity score matching. That was a 1:2 ultra-early to early group ratio, a 1:1 ultra-early to delayed group ratio, and a 3:1 early to delayed group ratio.
